# Polysome Profiling in Liver Identifies Dynamic Regulation of Endoplasmic Reticulum Translatome by Obesity and Fasting

**DOI:** 10.1371/journal.pgen.1002902

**Published:** 2012-08-23

**Authors:** Suneng Fu, Jason Fan, Joshua Blanco, Alfredo Gimenez-Cassina, Nika N. Danial, Steve M. Watkins, Gökhan S. Hotamisligil

**Affiliations:** 1 Department of Genetics and Complex Diseases and Department of Nutrition, Harvard School of Public Health, Boston, Massachusetts, United States of America; 2 Dana-Farber Cancer Institute and Harvard Medical School, Boston, Massachusetts, United States of America; 3 Broad Institute of Harvard and MIT, Boston, Massachusetts, United States of America; Ecole Polytechnique Fédérale de Lausanne, Switzerland

## Abstract

Obesity-associated metabolic complications are generally considered to emerge from abnormalities in carbohydrate and lipid metabolism, whereas the status of protein metabolism is not well studied. Here, we performed comparative polysome and associated transcriptional profiling analyses to study the dynamics and functional implications of endoplasmic reticulum (ER)–associated protein synthesis in the mouse liver under conditions of obesity and nutrient deprivation. We discovered that ER from livers of obese mice exhibits a general reduction in protein synthesis, and comprehensive analysis of polysome-bound transcripts revealed extensive down-regulation of protein synthesis machinery, mitochondrial components, and bile acid metabolism in the obese translatome. Nutrient availability also plays an important but distinct role in remodeling the hepatic ER translatome in lean and obese mice. Fasting in obese mice partially reversed the overall translatomic differences between lean and obese nonfasted controls, whereas fasting of the lean mice mimicked many of the translatomic changes induced by the development of obesity. The strongest examples of such regulations were the reduction in Cyp7b1 and Slco1a1, molecules involved in bile acid metabolism. Exogenous expression of either gene significantly lowered plasma glucose levels, improved hepatic steatosis, but also caused cholestasis, indicating the fine balance bile acids play in regulating metabolism and health. Together, our work defines dynamic regulation of the liver translatome by obesity and nutrient availability, and it identifies a novel role for bile acid metabolism in the pathogenesis of metabolic abnormalities associated with obesity.

## Author Summary

Chronic diseases including obesity and associated metabolic abnormalities have become the greatest threat to human health worldwide. How metabolic organs and organelles adapt to nutritional fluctuations, or fail to do so, remains incompletely understood. To explore these issues, we developed a new platform to explore translational responses in the liver, a critical organ for metabolic homeostasis. In this translatomic platform, we integrated polysome profiling and global analysis of polysome-associated mRNAs to systematically quantify protein synthesis on each transcript in obesity and during fasting. Our analysis demonstrated for the first time that protein synthesis is progressively suppressed in the obese liver and that the overall translatome profile of obese liver markedly resembles that of fasting lean mice, particularly in mitochondrial function and bile metabolism. We also examined the physiological impact of some of these alterations and concluded that aberrant bile acid metabolism in the obese liver represents a novel mechanism contributing to hyperglycemia and continuous weight gain. Together, our work reveals abnormal translational regulation as a novel aspect of obesity that could impact future directions in metabolic disease treatment, and we believe translatome profiling represents a new approach to unravel complex mechanisms regulating cellular function and disease pathology.

## Introduction

Proteins are the main workforces of a cell, and protein synthesis is highly controlled according to cellular needs. Cells modulate protein synthesis in response to hormonal cues, nutrient availability and episodes of intracellular stress. Anabolic hormones like insulin can stimulate the uptake of amino acids [1], the phosphorylation of ribosomal S6 protein, and the assembly of the eukaryotic initiation factor 4 (eIF4) complex to facilitate protein synthesis [2],[3],[4],[5]. Alternatively, nutrients, particularly L-branched amino acids, activate protein synthesis through the mammalian target of rapamycin pathway (mTOR) and its downstream effectors such as ribosomal protein S6 kinase (S6K1) and eukaryotic translation initiation factor 4 binding protein (eIF4BP1) [6],[7],[8],[9],[10]. Moreover, cells also modulate the rate of protein synthesis in response to stresses invoked by energy depletion, virus infection, and dysfunctional protein folding, through AMP-dependent protein kinase (AMPK) and multiple eukaryotic translation initiation factor 2 alpha (eIF2α) kinases, such as the interferon-induced, double-stranded RNA-activated protein kinase (PKR) and the PKR-like endoplasmic reticulum kinase (PERK) [11],[12],[13],[14]. Due to the fundamental role of protein synthesis in cellular function, mutations in the protein synthesis machinery and its regulatory pathways have been broadly implicated in many diseases [15].

Obesity is a critical risk factor for the development of systemic metabolic disease and also presents unique challenges to the protein synthesis machinery. On the one hand, obesity is a condition of over-nutrition, with characteristic elevations in plasma amino acid levels as well as hyper-activation of the mTOR pathway [16],[17]. On the other hand, obesity is typically associated with the development of insulin resistance, chronic inflammation and ER stress, and the level of eIF2α phosphorylation is uniformly increased, which would be expected to inhibit protein synthesis [18]. How these conflicting inputs are coordinated to modulate protein synthesis *in vivo* is largely unknown, and the effect of obesity and diabetes on protein metabolism seems to be tissue-dependent, and sometimes protein-specific [19]–[24]. However, the overall evidence suggests that muscle protein synthesis in obesity may be impaired at the basal and postabsorptive states, and the consequential loss of lean mass likely plays a role in the pathogenesis of diabetes by further impairing glucose and lipid disposal into skeletal muscle [24]. Conversely, stimulating protein synthesis through dietary amino acid supplementation (leucine in particular) has been found to result in a loss of adiposity and improvements in systemic glucose and lipid homeostasis [25]. Moreover, a genetic mouse model that promotes eIF2α dephosphorylation displays enhanced protein synthesis, resistance to diet-induced and genetic obesity, and improved glycemic control [26]. Together these studies suggest that while maintaining protein synthesis is crucial for systemic glucose and lipid metabolism, constitutive elevation of protein synthesis in obesity may also challenge homeostatic metabolism.

Liver is the main site for the production of glucose, cholesterol and very-low-density-lipoproteins (VLDL), perturbations of which commonly underlie the development of diabetes and cardiovascular complications precipitated by obesity. Liver also secretes copious amounts of plasma proteins that regulate immune response and peripheral tissue metabolism. However, the status of hepatic protein synthesis in animal models of insulin resistance is not well studied [19],[20]. To address this question, we performed polysome profiling and global analyses of polysome-bound transcripts in the liver of genetically obese mice under fasted and *ad libitum* fed states in comparison to lean controls. Our results demonstrated dynamic regulation of protein synthesis by states of obesity and nutrient availability, and revealed novel mechanisms linking bile acid metabolism to metabolic homeostasis in obesity.

## Results

### Suppression Of Er–Associated Protein Synthesis In The Obese Liver

**Figure 1:**
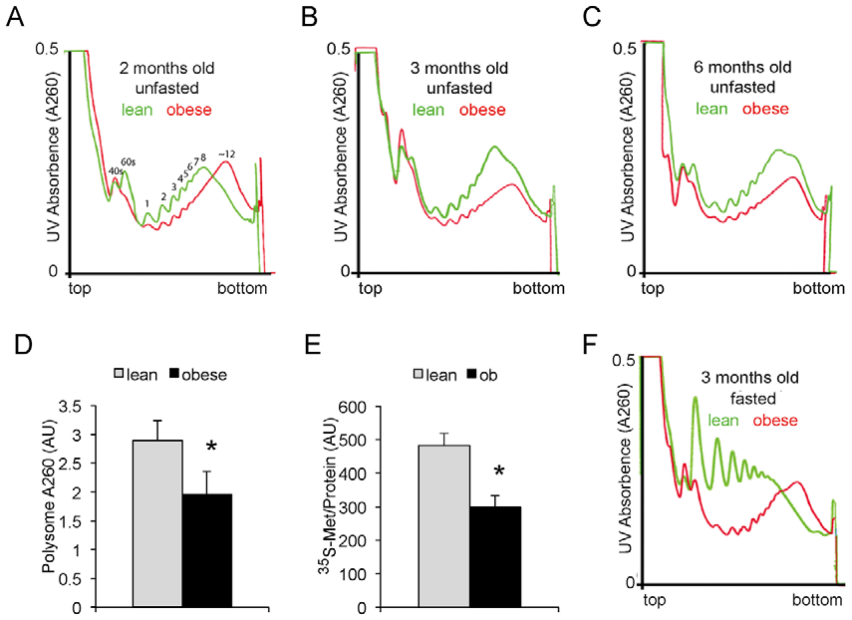
ER–associated polysome profiling of lean and obese liver tissues. (A) ER-associated polysome profile of lean and obese mouse liver tissues without overnight fasting. Membrane bound polysomes were released from ER and centrifuged through sucrose gradients to separate ribosomes according to their density with light ribosomes (ribosome subunits and single ribosomes) at the top and heavy ones (polysomes) at the bottom of the separation columns. The sucrose gradients were then fractionated from top to bottom and the concentration of ribosomes in each fraction was continuously monitored by UV absorbance (A260 for ribosomal RNA). The left to right of the X-axis correlates the top (light ribosome subunits and single ribosome) to the bottom (heavy, multiple ribosomes assembled on a single transcript) of the sucrose gradient respectively. The Y-axis A260 measures the total amount of ribosomal RNA present in each fraction. Ribosomes from equivalent amounts of ER were fractionated. (B–C) ER associated polysome profile of lean and obese mouse liver tissues at 3 and 6 months of age without overnight fasting. (D) Area under the curve measurements of the polysome fraction of lean and obese mouse livers at 3 months of age (n = 3, p<0.05, Student's *t*-test). (E) Quantification of *de novo* protein synthesis in lean and obese primary hepatocytes as measured by ^35^S-Methionine pulse-tracing (t = 30 minutes, n = 4, p<0.01, Student's *t*-test). (F) ER associated polysome profile of lean and obese mouse liver tissues at 3 months of age after overnight fasting. Equal amount of ER isolated from lean and obese mouse livers were loaded onto the sucrose gradient for polysome fractionation.

There are two main sites of protein synthesis in a cell: the cytosol and the endoplasmic reticulum (ER), with an additional small fraction of proteins synthesized in the mitochondrion. In this study, we have utilized polysome profiling to separate and quantify ribosome subunits and monosomes, which are not actively involved in protein synthesis, and polysomes, which have multiple ribosomes loaded onto single mRNA molecules and thus are an indication of active protein synthesis. Furthermore, comprehensive and quantitative analyses of polysome-bound transcripts allowed the identification and quantification of mRNA transcripts being actively translated, and it has been previously shown that the majority of proteins can be synthesized from both ER and cytosolic compartments, but membrane proteins and secreted proteins are almost exclusively synthesized in the ER-bound compartment [27]. Since obesity is characterized by ER dysfunction [18], we chose to profile ER-polysomes instead of cytosolic polysomes as they would provide more insights into organelle homeostasis as well as the secretory/systemic functions of the liver under the challenges of obesity. For this analysis, we fractionated and quantitatively determined the amount of ER-associated polysomes to evaluate the level of protein synthesis in the liver of lean and obese (*ob/ob*) mice. We found that the amount of membrane-bound ribosomes is dramatically reduced in the liver of obese mice (Figure 1A), consistent with our proteomic profiles that have shown down-regulation of ribosomal proteins associated with hepatic ER [28]. The reduction of protein synthesis on the obese ER is age dependent: in fact, younger mice (2 months) exhibited modestly higher levels of protein synthesis than wild type lean controls while older mice (3 and 6 months) exhibited progressively reduced ER-associated protein synthesis characterized by a decrease in the amount of mRNA bound, peptide synthesizing ribosomes (Figure 1B–1D). Direct measurement of protein synthesis using ^35^S-Methionine tracing experiments in isolated primary hepatocytes of lean and obese mice confirmed ∼50% reduction in overall protein synthesis in the obese liver (Figure 1E). However, despite the decrease in total ER-bound ribosomes in the obese liver, its overall ribosome profile was shifted toward polysomes, suggesting that, on average, there are more ribosomes bound to each translating mRNA molecule. It has been well established that the obese liver exhibits ER stress and is characterized by uniformly higher levels of eIF2α phosphorylation in both obese mouse models and in obese humans [29],[30], which should suppress protein synthesis by reducing the rate of translation initiation and the disassembly of polysomes to monosomes, as evidently displayed in lean, fasted mice (Figure 1F). However, the translational suppression in the obese liver is not associated with polysome disassembly. Instead, the overall polysome profiling of the obese mice was shifted to higher polysomes (Figure 1B and 1C), suggesting the contribution of an eIF2α phosphorylation-independent mechanism(s) in translational depression in the obese mice.

### Perturbation Of Liver Translatome By Obesity

**Figure 2:**
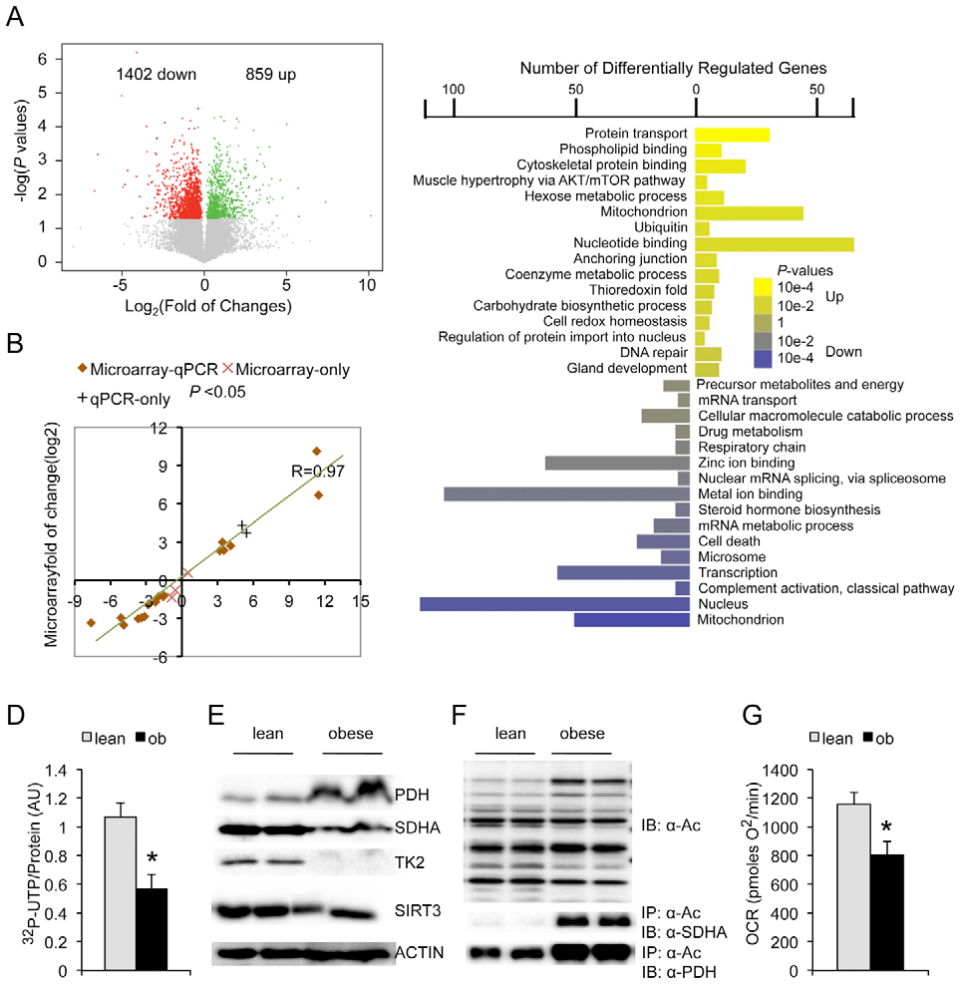
Genome-wide translational changes in the obese mouse liver. (A) Scatterplot of log*P*-values *versus* log_2_FC (fold of change, obese/lean). Green denotes genes up-regulated in the obese liver ER translatome while red denotes downregulation. (B) Differential regulation of transcripts as measured by microarray (Y-axis) and quantitative RT-PCR (X-axis) for 22 genes that were calculated as differentially regulated (student *t*-test, *P*<0.05) by either one or both methods. (C) Gene Ontology analysis of differentially regulated genes in the ER translatome of nonfasted lean and obese liver. X-axis denotes the number of genes categorized into each differentially-regulated function on the Y-axis. Color scale corresponds to log*P*-values converted to positive values for functional categories comprised of upregulated (obese/lean) genes. (D) Measurement of RNA synthesis in lean and obese primary hepatocytes based on ^32^P-UTP incorporation (t = 2 hours, n = 4, p<0.01, Student's *t*-test). (E) Immunoblot measurement of mitochondria protein expressions in the lean and obse mouse liver samples. (F) Immunoblot measurement of overall mitochondrial protein acetylation (IB: α-Ac, top panel) and the acetylation of the succinate dehydrogenase (IP: α-Ac; IB: α-SDHA, middle panel). Acetylation of the pyruvate dehydrogenase enzyme 1 (PDH-E1α) is shown (bottom panel) as a positive control for α-Ac immunoprecipitation as it is fully charged with acetyl-CoA under both lean and obese conditions. (G) Measurement of mitochondria oxygen consumption rate (OCR) for lean and obese primary hepatocytes (n = 7, p = 0.01, Student's *t*-test).

Systematic analyses of the polysome-bound transcripts were carried out to analyze potential alterations in the obese translatome, particularly preferential alterations in translated messengers, besides the overall decrease in ER-associated polysome content. A total of 2261 differentially regulated genes (1402 decreased and 859 increased in expression level, obese/lean, p<0.05) were identified between the obese liver ER translatome and that of lean controls, with many of them confirmed and validated by quantitative real time RT-PCR (Figure 2A–2B, Table S1). The most significantly regulated genes include those involved in bile acid synthesis (Cyp7b1, down in obese), bile acid transport (Slco1a1, down in obese), estrogen sulfotransferase (Sult1e1, up in obese) and cell death-inducing DNA fragmentation factor, alpha subunit-like effector A (Cidea, up in obese) (Table S1). Gene ontology analyses (DAVID, v6.7 [31]) revealed two prominent features associated with obese translatome (Figure 2C). First, we found that the synthesis of proteins involved in transcription, mRNA splicing and transport were all significantly downregulated (Figure 2C, Table S1). Indeed, measurement of RNA synthesis by ^32^P-UTP tracing in the primary hepatocytes isolated from lean and obese mice found significant reduction in the rate of UTP incorporation into nascent RNA molecules under the obese condition (Figure 2D). This concerted downregulation of transcription, mRNA splicing and transport functions in the obese translatome suggest a reinforcing mechanism deploying multiple devices to keep protein synthesis under control in obesity. Second, we observed strong dysregulation in the translation of many mitochondrial proteins (45 up, 48 down). Consistent with the overall down-regulation of protein synthesis in the obese liver, the synthesis of mitochondrial ribosomal proteins was also reduced in the obese translatome, suggesting a repression of mitochondrial protein synthesis. We also observed down-regulation of proteins in the mitochondrial electron transport complex but not those involved in the tricarboxylic acid (TCA) cycle (Figure 2E). The NAD-dependent deacetylase sirtuin 3 (SIRT3) has been shown to be a major regulator of mitochondria function by modifying the acetylation status of mitochondrial proteins [32]–[34]. We found that SIRT3 is reduced in the obese translatome, and overall mitochondrial protein acetylation is consequently increased in the obese liver (Figure 2E and 2F, Table S1). In particular, we found that the acetylation of succinate dehydrogenase (SDHA), a well-established substrate of SIRT3, was significantly increased despite the down-regulation of its total protein level (Figure 2F). Consistent with these analyses, mitochondria function is indeed reduced as measured by oxygen consumption rate in obese primary hepatocytes compared to matching lean controls (Figure 2G).

### Dynamic Regulation Of Liver Translatome By Food Deprivation

**Figure 3:**
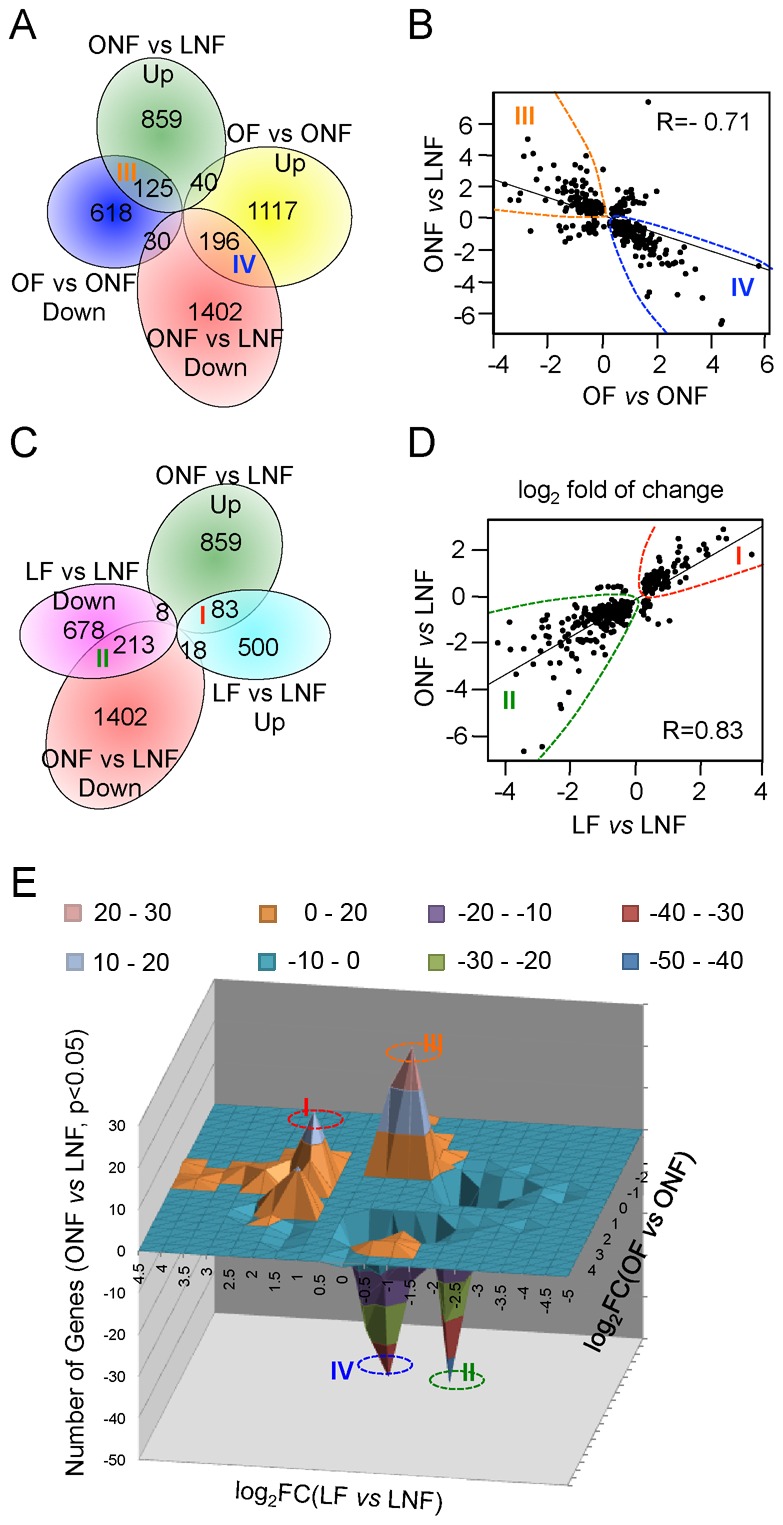
Dynamic regulation of the hepatic ER translatome by fasting. (A) Illustration of the overlap between translatomic changes induced by the development of obesity (obese nonfasted, ONF *versus* lean nonfasted, LNF) and fasting in lean mice (lean fasted, LF *versus* lean nonfasted, LNF). (B) Correlation scatterplot of fold changes (logarithmically transformed) for commonly regulated genes induced by obesity (Y-axis) and fasting in the lean (X-axis). Each dot represents a gene that is differentially regulated in the translatome in both comparisons (ONF *versus* LNF and LF *versus* LNF). (C) Diagram illustrates the reversal of obesity-induced translatomic changes (ONF *versus* LNF) by overnight fasting in the obese mice (OF *versus* ONF). (D) Scatterplot of logarithmically transformed fold changes induced by obesity (Y-axis) and its reversal by overnight fasting (X-axis). Each dot represents a gene that is differentially regulated in both comparisons (ONF *versus* LNF and OF *versus* ONF). “*R*” denotes Pearson coefficiency. (E) Distribution of commonly regulated genes between ONF *versus* LNF and LF *versus* LNF or OF *versus* ONF. X-axis: *P*-values of LF *versus* LNF; Y-axis: *P*-values of OF *versus* ONF; Z-axis: number of genes differentially regulated in the ONF *versus* LNF comparison. Negative values correspond to downregulated genes. I, genes upregulated in both ONF *versus* LNF comparison and LF *versus* LNF comparison; II, genes commonly downregulated between these comparisons; III, genes upregulated in ONF *versus* LNF comparison but downregulated in OF *versus* ONF comparison; IV, genes downregulated in the ONF *versus* LNF comparison but upregulated in the OF *versus* ONF comparison.

Obesity is traditionally considered a state of over-nutrition and dietary restriction is often the first line of treatment recommended to improve glucose control and induce weight loss. Interestingly however, in our studies we observed translational suppression, protein catabolism, and down-regulation of oxidative phosphorylation pathways in the obese translatome (Figure 1 and Figure 2), suggesting that obesity may, in some aspects, resemble a condition of nutrient deprivation at this site. This observation prompted us to comprehensively evaluate the relationship between obesity and nutrient regulation by systematic analysis of fractionated polysome-bound transcripts from lean and obese mice, comparing fasted and fed states. We found that overnight fasting altered the translation of ∼1700 genes, with nearly one quarter of these genes overlapping with the translatomic changes present between lean and obese nonfasted controls (Figure 3A). Importantly, an overwhelming majority of those changes (321 out of 391 genes) are in the opposite direction of those incurred by obesity (R^2^ = −0.71, Figure 3B), suggesting that a brief dietary restriction has the ability to improve a broad section of molecular defects associated with obesity. In particular, we found that SIRT3, which is down-regulated in the obese liver, was partially restored by fasting (Table S3). It has been previously shown that calorie restriction activates SIRT3 and reduces oxidative stress [35]. The observed upregulation of SIRT3 by overnight fasting in the obese liver suggests that regulatory mechanisms governing SIRT3 regulation are still intact, and the restoration of SIRT3 expression by overnight fasting may improve mitochondria function in the obese liver. Surprisingly, translatomic changes induced by overnight fasting of lean mice bore little or no resemblance to those that occurred in the obese mice, but mimicked many of the changes present in the obese, nonfasted liver (Figure 3C–3D). There is a large overlap between the gene sets regulated by fasting in the lean liver and those different between the lean and obese mice under nonfasted conditions: nearly a third (213 out of 678 genes) of the translatome down-regulated by fasting in the lean mouse liver overlaps with the non-fasted obese translatome as compared to the lean, and the direction and amplitude of regulation are extremely well correlated (R^2^ = 0.8, Figure 3D). As lean, overnight fasted mice bear little resemblance to obese fed mice regarding their nutritional status, the gene sets commonly regulated by obesity and fasting in the lean are likely downstream of some shared hormonal environments such as a lack of insulin signaling, enhanced glucagon action, and others that are yet to be defined.

### Regulation Of Systemic Glucose Homeostasis By Bile Acid Metabolism In The Liver

**Figure 4:**
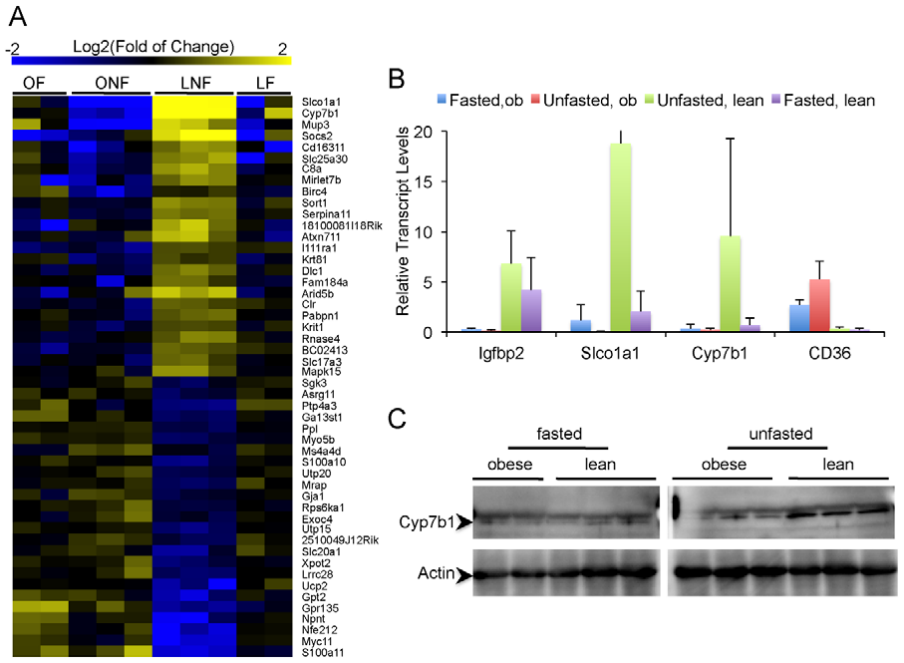
Dysregulation of bile acid metabolism in the obese ER translatome. (A) Heatmap of 50 most differentially regulated genes in the obese ER-associated translatome (25 up, 25 down) that are also suppressed in the lean fasted liver. ONF: obese non-fasted, LNF: lean non-fasted, OF: obese overnight-fasted, LF: lean fasted. Fold changes are calculated based on the mean of all ten samples. (B) Validation of feeding-induced upregulation of Cyp7b1 and Slco1a1, but not CD36 and Igfbp2, in the translatome of lean mouse liver. (C) Western blot of Cyp7b1 from the liver of lean and obese mice with or without overnight fasting.

**Figure 5:**
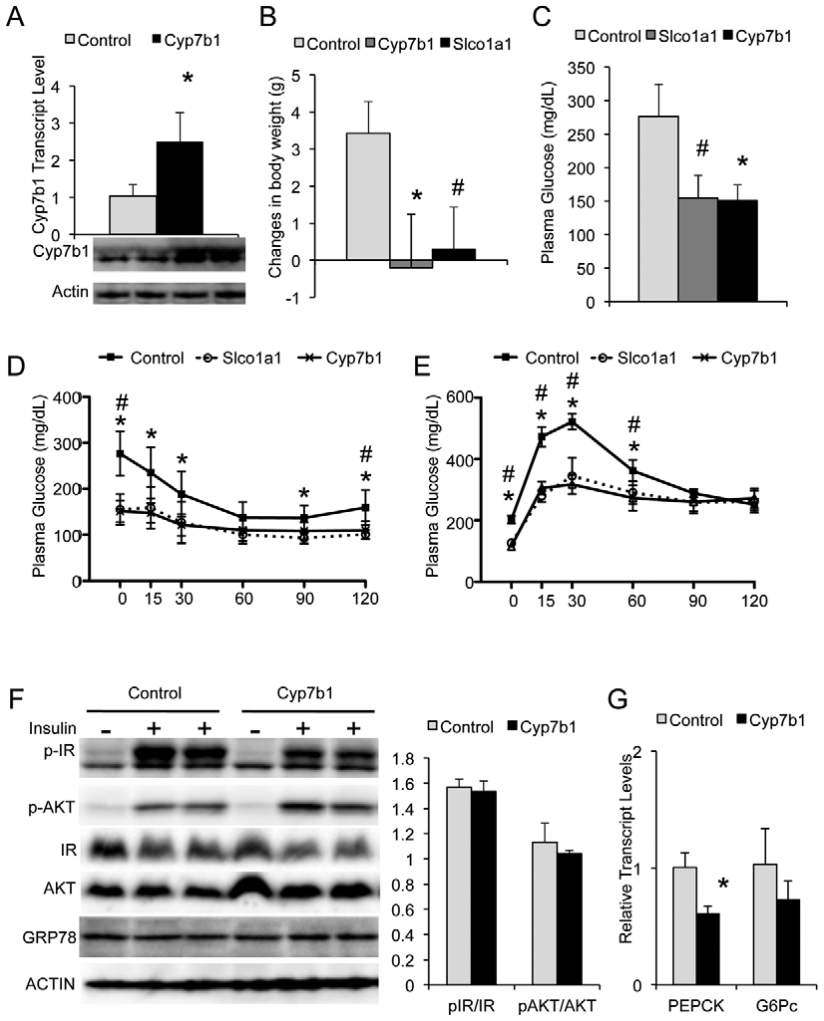
Cyp7b1 and Slco1a1 overexpression regulates glucose homeostasis. (A) Examination of Cyp7b1 expression in the liver of *ob/ob* mice transduced with control and Cyp7b1 over-expressing adenoviruses by quantitative RT-PCR and immunoblot analysis. (B) Changes of body weight in control and Cyp7b1 and Slco1a1 overexpressing mice in a 14 day period post-virus injection. (C) Glucose levels of control and experimental mice 5 days post-virus injection. (D–E) Insulin and glucose tolerance test of control and experimental mice with bolus injections of insulin (1.0 IU/kg) or glucose (0.75 g/kg) and measurement of plasma glucose levels at indicated times (n = 9). (F) Immunoblot analysis of insulin signaling stimulated by portal vein injection as indicated by insulin receptor (IR) and AKT phosphorylation. The graph on the right side shows the quantitation of the data after correction for total protein amount. (G) Expression of gluconeogenic genes in the liver of control and experimental mice as measured by with real time, quantitative RT-PCR (n = 4). “*” denotes p<0.05 comparing control versus Cyp7b1-overexpressing mice, and “#” denotes p<0.05 comparing control versus Slco1a1 overexpression, Student's *t*-test. PEPCK: phosphoenolpyruvate carboxykinase; G6Pc: Glucose-6-phosphatase catalytic subunit.

It came to our attention that the genes most down regulated by obesity and fasting, as compared to the lean-non-fasted state, are involved in bile acid metabolism. The most prominent examples included Cyp7b1, which catalyzes the synthesis of bile acid from cholesterol through the acidic pathway and was downregulated by 32-fold, and Slco1a1, a transporter involved in the reuptake of bile acids into the liver and that was suppressed by 100-fold (Figure 4A, Tables S1 and S2) [36],[37],[38]. This pattern of regulation is consistent with our previous ER proteomic studies [28] and validated by transcript as well as immunoblot analysis performed in independent liver samples (Figure 4B–4C). The dramatic induction of these two genes in lean-fed mice, combined with their defective expression in obese animals suggests a postprandial mechanism involving bile acid metabolism that is defective in the obese, insulin-resistant state. To test this hypothesis, adenovirus mediated overexpression experiments were carried out to uncover the biological relevance of the detected alterations in insulin-mediated regulation of bile acid metabolism and how its defects may contribute to metabolic abnormalities in obesity (Figure 5 and data not shown). Transient overexpression of Cyp7b1 and Slco1a1 reduced weight gain in obese mice and normalized plasma glucose levels (Figure 5A–5C). This improvement in systemic glucose homeostasis, also evident in glucose tolerance tests (Figure 5D) was also reflected in the glucose excursion profile in insulin tolerance tests, although here the blood glucose levels at the onset were already markedly reduced thus the excursion curves were less dynamic (Figure 5E). There was little further reduction in blood glucose from this low baseline value upon insulin administration, suggesting these mice may not exhibit a significant alteration in insulin sensitivity *per se*. Insulin receptor and Akt phosphorylation per protein in the liver tissue were similar between groups (Figure 5F). Taken together, these results suggest that the decrease in plasma glucose levels upon exogenous expression of Cyp7b1 in obese mice may be primarily driven by regulation of glucose output, correlated with the downregulation of the gluconeogenic gene expression (Figure 5G).

### Regulation Of Hepatic Steatosis And Cholestasis By Cyp7b1 Overexpression

**Figure 6:**
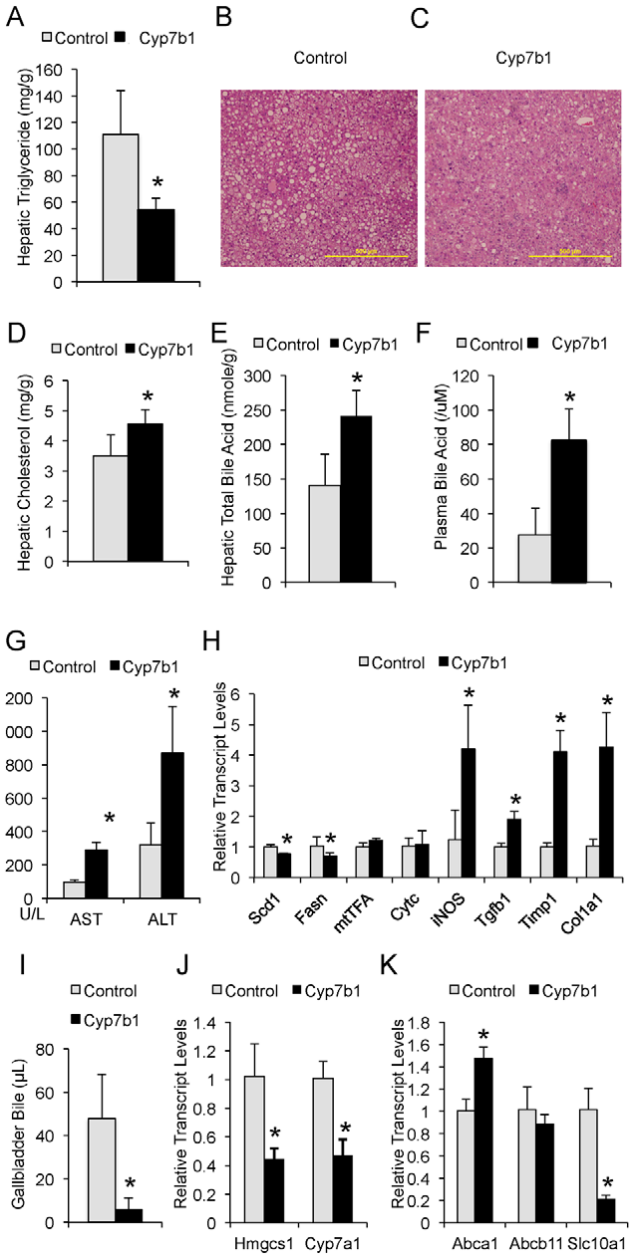
Cyp7b1 overexpression causes cholestasis. (A–C) Changes of hepatic steatosis in the liver of control and experimental mice as measured by liver triglyceride levels and H&E staining. White vesicles indicate fat infiltration. (D–E) Measurement of hepatic cholesterol and bile acid levels in the control and experimental mice. (F–G) Measurement of plasma ALT/AST levels and bile acid levels from the control and experimental mice. (H) Transcript levels of genes involved in inflammation and fibrosis in the liver of control and experimental mice as measured by qPCR. (I) Measurement of total bile collected from the gallbladder of control and experimental mice after 6 hours of food withdrawal. (J–K) Measurement of transcript levels of genes involved in cholesterol and bile synthesis and transport in the liver tissues of control and experimental mice with real time, quantitative RT-PCR. “*” denotes p<0.05 (Student's t-test, n = 6 except for qPCR, in which n = 4/group).

We carried out additional histological and biochemical characterization of the Cyp7b1-overexpressing *ob/ob* mice to decipher the mechanism underlying the improved basal glucose levels but not insulin sensitivity. Cyp7b1-overexpressing mice exhibited improved hepatic steatosis as measured by total triglyceride levels and haemotoxylin Eosin (H&E) staining (Figure 6A–6C). On the other hand, both hepatic cholesterol and bile acid levels were increased in these mice (Figure 6D and 6E). The development of hepatic cholestasis was accompanied by liver injury as evident from the elevation of plasma bile acid levels, aspartate aminotransferase (AST) and alanine aminotransferase (ALT) levels, as well as the upregulation of inflammatory and fibrosis genes (iNOS, Tgfb1, Timp1, Col1a1) in the Cyp7b1-overexpressing liver (Figure 6F–6H). The development of cholestasis in the Cyp7b1-overexpressing *ob/ob* liver was not caused by increased cholesterol biosynthesis and bile formation. In fact, bile formation was greatly reduced in the Cyp7b1-overexpressing mice, as measured by gallbladder bile volumes (Figure 6I). Consistent with the reduction in bile formation, the expressions of rate limiting enzymes involved in cholesterol and bile acid biosynthesis (Hmgcs1 and Cyp7a1, Figure 6J) were both downregulated. The development of cholestasis is also not caused by dysregulation in bile acid export or enterohepatic reuptake. As shown in Figure 6K, the expression of bile salt export pump (BSEP/Abca1) was upregulated in the Cyp7b1 overexpressing liver, while Na+-taurocholate cotransporting protein (NTCP/Slc10a1), the main transporter required for bile acid reuptake into the liver, was significantly downregulated. Taken together, these results suggest the downregulation of Cyp7b1 in the *ob/ob* liver has an unexpected role in maintaining bile formation. Forced overexpression of Cyp7b1 improved hepatic steatosis but eventually suppressed bile acid excretion and caused cholestasis.

## Discussion

By combining polysome profiling with microarray analyses, we for the first time established a mammalian translatome *in vivo*. The translatome analysis established herein is distinct from recently published works based on deep sequencing of ribosome footprints on mRNA [39],[40]. As microarray platforms have been broadly established, we believe our method offers a cost-effective alternative to the aforementioned technology. Integration of translatomic analyses with transcriptomic and proteomic analyses in the future will greatly improve our understanding of the molecular mechanisms governing cell biology and disease pathology by distinguishing transcriptional, translational and post-translational events.

Our analysis revealed several important insights that should serve as the basis for future experimentation and other mechanistic studies, as well as potential novel therapeutic strategies. One such concept emerging from this work is that in obesity, liver experiences a molecular fasting profile despite the presence of abundant nutrients, suggesting the presence of peripheral systems that govern hunger response. As obesity may represent a similar hormonal environment to the lean fasted state (e.g. insulin resistant *versus* insulin absent), we suggest that the gene set that is commonly regulated by lean fasting and obesity harbors novel mechanisms linking insulin resistance to the pathogenesis of metabolic disorders including diabetes and cardiovascular disease. On the other hand, the gene set reversed by overnight fasting in the obese translatome may represent cellular networks that are mainly responsive to nutritional signals, as overnight fasting is unlikely to significantly alter the status of insulin signaling (and probably other major hormones too) in the obese liver. Future work will need to decipher whether and how these genes may contribute to the development of overnutrition-induced obesity and insulin resistance or other metabolic responses.

Another important and related finding here is the discovery of novel regulatory patterns governing bile acid metabolism. Bile acids have long been recognized as an important player in facilitating the absorption of lipids in the small intestine, and catabolism of cholesterol to bile acid accounts for ∼50% of daily cholesterol turnover [41]. More recently, evidence has emerged suggesting that bile acids also function as hormones that regulate systemic glucose and energy homeostasis [42]–[45]. However, the role of bile acid metabolism in the pathogenesis of metabolic syndrome is not well studied. Our results in this study suggest that the Cyp7b1-catalyzed, alternative bile acid synthesis pathway is a negative regulator of gluconeogenesis and hepatic triglyceride accumulation, and this pathway is defective under fasting or insulin resistant conditions. Thus, defective Cyp7b1 expression may be a component of obesity-related metabolic pathologies including hyperglycemia and hepatic steatosis. However, caution has to be exercised concerning the role of Cyp7b1 in metabolic regulation, because constitutive overexpression of Cyp7b1 also led to the development of cholestasis. The overall changes in the gene expression patterns of Cyp7b1-overexpressing liver related to gluconeogenesis, lipogenesis, cholesterol and bile acid synthesis and transport are consistent with bile acid-mediated activation of FXR and SHP [42],[46],[47], and CDCA, the main product of the Cyp7b1 pathway, is the most potent agonist of FXR [46]. However, the accumulation of cholesterol and bile acid in the Cyp7b1 overexpressing liver is not due to increased synthesis, as Cyp7b1 contributes to only ∼5% of total bile production in normal physiology [48],[49], and the main bile acid synthesis pathway is suppressed 50% (Figure 6J). We suggest that extended overexpression of Cyp7b1 in the obese mouse liver has metabolic benefits but also carries risks by altering hepatic bile acid and lipid excretion. However further studies are warranted to explore the details of the mechanism and reconsider the potential of bile acid-based approaches for the treatment of diabetes and other metabolic diseases associated with obesity [50],[51].

Taken together, our experiments demonstrated tissue polysome profiling as a powerful approach to explore novel mechanisms underlying homeostatic responses that are dynamically regulated in response to nutrient intake and disease pathology, which would not otherwise be apparent. Application of this platform to other metabolically critical organs may provide additional insights into disease mechanisms. In particular, muscle exhibits strong defects in protein metabolism and mitochondrial dysfunction [21],[52]–[56]. It will be extremely interesting to study how the translatome profile of obese muscle tissue is different from its lean counterparts and how these differences may contribute to muscular dystrophy and glucose intolerance in diabetic patients.

## Materials and Methods

### Ethics Statement

Animal use and care in this study strictly follows the Guidelines For The Use Of Non-Human Vertebrate Animals In Research And Teaching, as established by Harvard University's Institutional Animal Care and Use Committee (IACUC).

### Animals

Homozygous male leptin deficient (ob/ob) and wild type littermates in the C57BL/6J background were either bred in house or purchased from Jackson Laboratories (000632). All mice were maintained on a 12-hour-light/12-hour-dark cycle in a pathogen-free barrier facility with free access to water and regular chow diet (Rodent Diet 5058). Mice were sacrificed at three months of age for polysome fractionation unless otherwise noted. All animal experimental procedures were approved by the Harvard University IACUC.

### Er–Associated Polysome Profiles

The protocol for separating cytosol and ER-bound polysomes in liver tissue was adapted from Ramsey and Steele [57]. Briefly, mice with or without overnight fasting were anesthetized by tribromoethanol and perfused with 20 ml of 0.25M sucrose and 1 mM MgCl_2_ solution. Livers were excised, homogenized in 6 times the volume of 0.25M sucrose/HKMG buffer (50 mM HEPES pH 7.2, 75 mM KCl, 5 mM MgCl_2_, 3 mM Glutathione), and centrifuged to obtain an ER pellet. The ER pellet was then resuspended in 33% ribosome free cell sap. The membrane bound polysomes were released into the supernatant by stepwise addition of Triton X-100 and sodium deoxycholate to 1% followed by centrifugation at 13,000 rpm for 10 min to pellet all membrane material. Equal amount of microsomes in a total volume of 200 µl were separated onto 11 ml of 10–50% sucrose gradient by centrifugation at 35,000 rpm for 3 hours using SW41 rotor. The sucrose gradient was then fractionated and UV absorption at 260 nm was recorded.

### Isolation Of Polysome-Associated Rna And Quantitative Real-Time Rt–Pcr

Sucrose fractions containing >3 polysomes were pooled and total RNA was extracted with 3 volumes of Trizol LS (Invitrogen,CA) and precipitated with isopropanol and high salt solution (0.8M Sodium Acetate+1.2 M NaCl) according to the manufacturer's recommendations. A total of 2 µg of RNA was used for cDNA synthesis using High Capacity cDNA archiving kit (Applied Biosystems). The SYBR real-time PCR system was used to quantify transcript abundance for genes of interest (Table S4). Either 18S or 28S rRNA was used for internal control.

### Rna Preparation For Affymetrix Microarray Analysis

For each experimental sample, RNA quality was assessed by Agilent Bioanalyzer 2100 RNA Nano LabChip analysis system. Under standard conditions processing of RNAs for GeneChip Analysis was in accordance with methods described in the Affymetrix GeneChip Expression Analysis Technical Manual (rev. 1.3), as subsequently detailed. Synthesis of cDNA first and second strand is performed using the GeneChip Expression 3′-Amplification Reagents One-Cycle cDNA Synthesis system (P/N 900431). Cleanup of the double stranded product is carried out according to standard Affymetrix protocols using the Affymetrix GeneChip Cleanup Module (Affymetrix Catalog # 900371). In vitro transcription (IVT) is performed using the GeneChip Expression Amplification Reagents system- 30 reactions (P/N 900449) and is carried out according to the standard Affymetrix protocols. Quantification of the IVT samples is carried out on a Bio-Tek UV Plate Reader.

### Microarray Hybridization, Scanning, Normalization, And Annotation

Hybridization is carried out according the Affymetrix GeneChip Manual (rev. 1.3). Twenty micrograms of IVT material is the nominal amount used on the GeneChip arrays. Affymetrix hybridization ovens are used to incubate the arrays at a constant temperature of 45°C overnight. Preparation of microarrays for scanning is carried out with appropriate Affymetrix wash protocols matched to the specific chip type on a Model 450 Fluidics station. Affymetrix GeneChip Operating Software (GCOS) operating system controls the Fluidics station process. Scanning is carried out on an Affymetrix Model 3000 scanner with autoloader. The Affymetrix GCOS operating system controls the scanner and data acquisition functions. GCOS maintains the mediated first level data analysis and desktop data management for the entire GeneChip System. Chip library files specific to each array and necessary for scan interpretation are stored on the computer workstation controlling the scanner and are updated regularly as necessary when updates are made available from Affymetrix. Subsequent data analysis was carried out using the analysis tools contained in GenePattern. Microarray data were deposited in the public Gene Expression Omnibus (GSE39375).

### Statistical And Bioinformatic Analysis

A variety of software packages were applied to identify differentially regulated genes from the microarray including SAM, LIMMA, DEDS as well as Student's *t*-test. The result of Student's *t*-test was presented herein as it is most consistent with quantitative RT-PCR validations. Gene Ontology analysis for differentially regulated genes was carried out with the database for annotation, visualization and integrated discovery (DAVID, v6.7, [31]). Heatmap was generated in MeV [58]. Graph and charts of statistical analyses were plotted in R.

### Primary Hepatocyte Isolation

For the isolation of primary hepatocytes, lean and obese mice were anesthetized by ketamine/xylazine, perfused with HBSS buffer (Invitrogen,CA) supplemented by 1 mM EGTA and 5 mM of glucose and then digested with Collagenase X(WAKO, Japan) at 0.5 mg/mL dissolved in HBSS buffer supplemented with 1.2 mM CaCl_2_ and 5 mM glucose. Primary hepatocytes released by collagenase digestion were sedimented at 50 g for 1 minute and washed for three times with medium 199 (Invitrogen, CA). The viability of cells was checked by tryptophan blue (Invitrogen, CA).

### Measurement Of Protein And Rna Synthesis In Primary Hepatocytes

For the measurement of protein and RNA synthesis in the lean and obese primary hepatocytes, equal numbers (2×10^5^/well) of cells were plated on 12-well BioCoat plates (BD Bioscience, MA) for overnight. Cells were then washed in PBS for three times, re-cultured in DMEM minus methionine supplemented with 10% dialyzed FBS for half an hour to depleted internal stores of methionine, and then pulse chased with either ^35^S-Methionine or ^32^P-UTP in the same medium for another 30 minutes. Cells were washed three times with PBS, re-cultured in regular DMEM+10% dialyzed FBS for either 30 minutes (for measuring protein synthesis) or 2 hours (for measuring RNA synthesis). Upon termination of the experiment, cells were lysed, protein and RNA content were extracted and precipitated with either acetone (protein) or ethanol (RNA), resuspended in water and their radioactivities were measured. Protein and RNA synthesis rates were normalized to total cellular proteins prepared from parallel experiments without radioactive labeling.

### Mitochondria Oxygen Consumption In Primary Hepatocytes

Mitochondrial oxygen consumption rate (OCR) was measured in real time in whole hepatoytes using the XF24 extracellular flux analyzer from Seahorse Bioscience [59]. Hepatocytes were seeded on collagen-coated XF24 V7 plates (Seahorse Bioscience, MA) at 4×10^4^ cells/well, allowed to attach for at least 2 hours, rinsed once and kept in 600 µl of sodium bicarbonate-free DMEM medium (Sigma, MO) supplemented with 10 mM glucose and 1 mM sodium pyruvate. After baseline measurements, the complex I inhibitor rotenone (Sigma, MO) was injected at 1 µM final concentration to determine the total mitochondrial oxygen consumption. Parallel samples were run in the absence of any treatment to ensure stable baselines as a quality control parameter for the bioenergetic health of the cells. The respiratory rate was measured at 37°C in 8 replicates (independent wells) for each of the seven pairs of lean and obese mice. Rates were calculated and analyzed using the Seahorse XF24 v1.7.0.74 software.

### Adenovirus-Mediated Gain-Of-Function Experiments

The open reading frames (ORF) of mouse Cyp7b1 and Slco1a1 were amplified, cloned into the pENTR/D-TOPO vector, and then recombined into the pAD/CMV/V5-DEST vector. Adenoviruses for constructs of interest were produced and amplified in 293A cells, purified using CsCl column, desalted, and 0.15 OD of virus particles were used for each mouse. Blood glucose levels were measured after 6 hours of food withdrawal (9am–3pm) at 5 days post-virus injection and at the time of harvest (9–12 days). Insulin and glucose tolerance test were performed with a bolus, intraperitoneal injection of insulin (1 IU/kg) or glucose (0.75 g/kg) and plasma glucose levels were measured every 15 or 30 minutes. The measurement of hepatic triglyceride (Sigma, MO), cholesterol (WAKO, VA) and bile acids (Cayman Chemicals, MI) as well as plasma bile acid content and serum alanine aminotransferase (ALT) and aspartate aminotransferase (AST) levels were made according to manufactures' recommendations (Cayman Chemicals, MI and BioVision, CA). For histological analysis, liver tissues were fixed in 10% formalin solution, and sectioned for Hematoxylin and Eosin (H&E) staining.

### Protein Lysate Preparation And Western Blot Analyses

Protein lysate preparation and immunoblot analyses were carried out essentially as described by Furuhashi et al [60]. Briefly, following 6 hours of food-withdrawal, *ob/ob* mice were euthanized in CO_2_ chambers and tissues of interest were collected and snap frozen in liquid nitrogen. Protein lysates were prepared in a cold lysis buffer containing 50 mM Tris-HCl (pH 7.0), 2 mM EGTA, 5 mM EDTA, 30 mM NaF, 10 mM Na_3_VO_4_, 10 mM Na_4_P_2_O_7_, 40 mM β-glycerophosphate, 0.5% NP-40 and 1% protease inhibitor cocktail, homogenized with polytron homogenizer, and followed by two steps of centrifugation (3000 rpm, 10 minutes and 13,000 rpm, 10 minutes). Total protein contents were measured by Bradford method (Bio-Rad, CA), and equal amounts of protein were subjected to SDS–polyacrylamide gel electrophoresis, transferred to 0.2 µ PVDF membranes (Whatman) and blocked with either 5% BSA or non-fat milk in TBS buffer containing 0.1% Tween 20. Antibodies used in this study include: AKT1/2/3, p-AKT1/2/3, ATP5J, IRβ, NDUFV2, PDH-E1α, SDHA, TK2 (Santa Cruz), CYP7B1 (ABNOVA), Acetylated proteins, SIRT3 (Cell Signaling), p-IRβ (Calbiochem) and Actin (Amersham Biosciences). Immunodetection analyses were accomplished using the enhanced chemiluminescence system (Roche Diagnostics). For immunodetection of acetylated SDHA and PDH-E1α proteins, a total of 5 mg proteins were incubated with 10 µg of anti-Acetyl-Lysine antibodies (Cell Signaling) and pulled down with 40 µl of Dynabeads (Invitrogen). Five percent of the elution is loaded onto SDS-PAGE for the detection of target proteins.

## Supporting Information

Table S1 List of all 2261 genes/probes differentially regulated in the lean and obese ER translatome. (XLS)

Table S2 List of 296 genes/probes commonly regulated under lean-fasting condition and obesity. (XLS)

Table S3 List of 321 genes/probes that are differentially regulated by obesity but corrected by overnight fasting. (XLS)

Table S4 List of oligos/probes used in this study. (XLS)
